# Real-time gigahertz free-space quantum key distribution within an emulated satellite overpass

**DOI:** 10.1126/sciadv.adj5873

**Published:** 2023-12-01

**Authors:** Thomas Roger, Ravinder Singh, Chithrabhanu Perumangatt, Davide G. Marangon, Mirko Sanzaro, Peter R. Smith, Robert I. Woodward, Andrew J. Shields

**Affiliations:** Toshiba Europe Ltd., 208 Cambridge Science Park, Cambridge, UK.

## Abstract

Satellite quantum key distribution (SatQKD) intermediated by a trusted satellite in a low-Earth orbit to ground stations along the satellite’s path allows remote users to connect securely. To establish a secure connection, a SatQKD session must be conducted to each user over a dynamically changing free-space link, all within just a few hundred seconds. Because of the short time and large losses under which the QKD protocol will be implemented, it has not yet been possible to form a complete key by transmitting all the relevant information required within a single overpass of the satellite. Here, we demonstrate a real-time QKD system that is capable of forming a 4.58-megabit secure key between two nodes within an emulated satellite overpass. We anticipate that our system will set the stage for practical implementations of intercontinental quantum secure communications that can operate over large networks of nodes and enable the secure transmission of data globally.

## Teaser

An experimental emulation of a single pass, satellite quantum key distribution system delivering real time secure symmetric keys.

## INTRODUCTION

Although secure quantum communications have been implemented commercially at the metropolitan (*1*–*3*) and national level (*4*), currently available technology is limited to operate over direct link distances in the region of ⁓100 to 1000 km (*5*, *6*). This is because the quantum signal cannot be noiselessly amplified in the same manner as classical communications (*7*, *8*). Despite the promise of quantum repeaters (*9*–*11*) to overcome this direct transmission limitation (*12*–*15*), the technology is some way off achieving the required performance to service an intercontinental quantum internet (*16*, *17*).

Recently, quantum key distribution (QKD) has been performed from a low-Earth orbiting (LEO) satellite to ground stations and has been shown to provide secure data transfer between nodes separated by intercontinental distances (*18*). In these tour-de-force demonstrations, large apertures were used at the satellite and ground stations to minimize the losses experienced by the quantum signal (*18*–*20*). For instance, in (*19*), the channel loss was just 19.5 dB, which enables the use of low clock rates, i.e., ≤200 MHz. The use of large telescopes allowed the satellite to transmit a large number of quantum bits (qubits) to the optical ground station within the limited time window of the overpass. However, radio frequency (RF) communication was used to provide the additional service channel for reconciliation and key distillation processes required by the QKD protocol. While this enabled the distribution of a sufficient number of qubits to form a positive quantum key, they were unable to distill the final secret key within a single overpass due to the limited bandwidth of the RF communication channel (particularly the uplink) (*19*, *21*). Specifically, the communicating parties were unable to perform sifting (key reconciliation) within the given time period. Data relevant to the quantum key must therefore be stored by the satellite until subsequent passes are possible, which can be of the order of terabytes in size (*22*). This fundamentally limits the number of ground nodes the satellite can communicate with due to limited storage on board the satellite platform. Furthermore, quantum keys are not ready for use until the full protocol has been completed, which may take multiple subsequent passes depending on the QKD system parameters and specific satellite trajectory under consideration.

One challenge facing satellite QKD (SatQKD) arises because of finite key size effects that become significant due to the relatively short key strings that are generated within a satellite overpass (*22*, *23*). To ensure that the keys at the quantum transmitter (QTx) and receiver are matching and secure, processes known as error correction (EC) and privacy amplification (PA) are applied (*24*). Following these processes, the raw key string will be reduced by a factor that is dependent on the error rate and system parameters. In terrestrial QKD links, where operation is continuous, long keys can be input to PA, and the output secure key can approach the asymptotic limit (*25*). This is the theoretical maximum output key length, which is derived assuming input keys of infinite length. However, for very short strings input to PA, the key is reduced by a larger amount due to statistical uncertainties. For particularly short key strings, the PA process may result in no output key at all (*22*). To overcome this, SatQKD systems should be designed to maximize the transmission efficiency between transmitter and receiver. This is costly because of the price of telescopes scaling approximately exponentially with aperture size (*26*). For more modest and commercially viable aperture sizes, one may instead look to increase the number of qubits transmitted within the given time window. This can be achieved by increasing the clock rate of the system from hundreds of megahertz to gigahertz. However, this also increases the amount of classical data that are required to be communicated between the satellite and ground station. For radio communication, this is extremely challenging because of the inherently large beam divergence of RF comms and low receiving power of the antenna on small satellite platforms, such as CubeSats or Microsats (*27*). Laser communications (lasercomms), however, can easily provide the bandwidth required to transmit all the relevant information within the temporal window of the overpass (*28*, *29*).

In this article, we present a gigahertz real-time QKD system that has been designed to complete the entire QKD protocol within a single satellite overpass. We emulate a satellite overpass assuming realistic and scalable dimensions for the transmitter and receiver telescope apertures and for an orbit altitude of 500 km. The system integrates a QTx seeded by a high-rate quantum random number generator (QRNG), a quantum receiver (QRx), classical communications via lasercomms, and pointing and tracking (PAT) via fast-steering mirrors (FSMs) in closed-loop feedback using beacon lasers. In the following sections, we define the details of the emulated overpass, describe the system hardware, and show the performance of our system throughout the overpass.

**Fig. 1.**
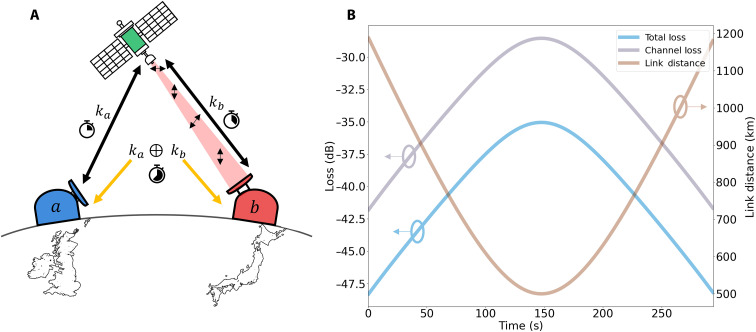
Example satellite-to-ground QKD scenario. (**A**) Schematic of a possible SatQKD protocol. A LEO satellite performs a QKD session with user *a* to form a key *k_a_* and a second QKD session with user *b* forming a key *k_b_*. The XOR of the two generated keys is publicly announced by the satellite to the two ground sites *k_a_* ⊕ *k_b_*. Users can then reconcile the key of the other user using the public ciphertext and their unique QKD key (*k*_*a*,*b*_). (**B**) Simulation of the channel loss, total loss, and link distance for a typical SatQKD session with a satellite trajectory with maximum elevation to the ground site of 90° and starting and stopping at an elevation of 20° and 160°, respectively.

A method to allow quantum secure communication between two ground nodes intermediated via a LEO satellite link is shown in Fig. 1A (*7*). A satellite hosting a QTx performs a QKD session with the first user *a*, who receives the QKD signal using a QRx and generates a secure quantum key *k_a_*. The satellite ends this QKD session, and a new session is performed with user *b*, forming a second unique quantum key *k_b_*. The satellite can then broadcast publicly the exclusive OR (XOR) operation of the two distributed keys *k_a_* ⊕ *k_b_*. By performing the XOR operation of the key from their unique session with that of the publicly broadcast ciphertext, the two users, *a* and *b*, may retrieve the key of the other user, i.e., *k_b_* = *k_a_* ⊕ (*k_a_* ⊕ *k_b_*). In this work, we consider how our system performs during a typical SatQKD session, i.e., to distribute keys from the satellite to a ground user. To do this, we first must estimate the expected losses.

We model the expected transmission efficiency of a satellite overpass by estimation of the channel losses through a number of different processes. These include diffraction, atmospheric absorption, pointing error, turbulence effects, the efficiency of optical components at the ground station, and the single-photon detection efficiency. The transmission efficiency per unit time of the satellite overpass is used to set the channel loss in our laboratory experiments. We have explored our system’s functionality over many different passes whereby the maximum elevation is varied. We start by considering the “optimal” overpass, whereby the satellite passes directly overhead with an elevation at closest approach of 90° and consider that the QKD system is operational between elevation angles of 20° and 160°, resulting in a total time of the overpass *t*_pass_ ≈ 294 s. The system performance for passes with maximum elevation <90° is summarized in Discussion and is shown in detail in the Supplementary Materials. When all the losses are considered together, we estimate the average channel loss (resulting from diffraction, atmospheric absorption, and turbulence) for the quantum downlink to be 34.00 dB during the optimal overpass. The channel loss, the total loss (which also includes the detection efficiency and loss due to collection optics), and the link distance are plot as a function of time (*t*_pass_) in Fig. 1B. Detailed parameters of the overpass, including the uplink and downlink losses for the auxiliary channels, which are taken into account in our experiments, can be found in Materials and Methods and the Supplementary Materials. The passes explored in this study assume nighttime operation to achieve the expected losses, and the parameters of passes operating in the day will be the subject of future studies.

**Fig. 2.**
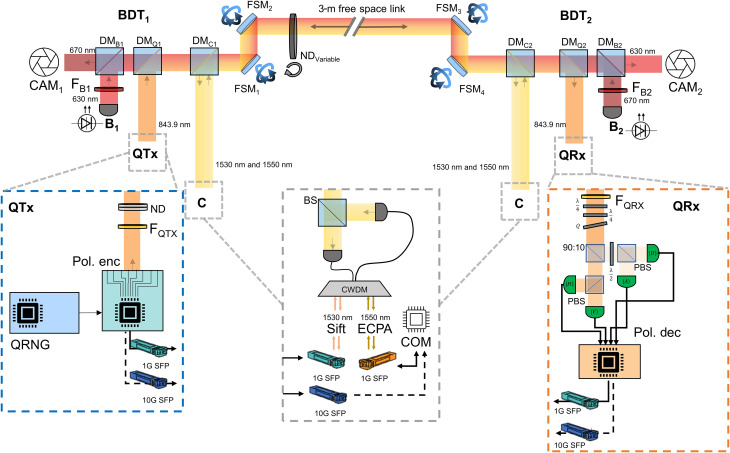
Experimental setup to emulate a satellite overpass within the laboratory Two BDTs (BDT_1,2_), consisting of a series of DMs (DM_*B*,*Q*,*C*_), are used to multiplex/demultiplex the beacon lasers (B_1,2_), the bidirectional classical communications lasers (C), and the quantum signal at the QTx and QRx. The polarization decoder at the QRx consists of a series of wave plates (λ/4, *Q*) to align the decoding reference frame to that of the transmitter, and the measurement basis is passively selected by a 90:10 BS (90:10). The four polarization states are measured by four single-photon detectors via PBSs with a half–wave plate (λ/2) used to rotate one of the detector pairs into the diagonal, anti-diagonal polarization (minority) basis. The QTx is seeded by a QRNG that is used to generate the quantum key in real time. The beacon and quantum signals are optically filtered (F_*B*_1_,*B*_2_,*QTX*,*QRX*_) to increase the signal-to-noise ratio. Two FSMs are used to misalign the system with the expected pointing error. A further two FSMs are used in closed loop with the beacons and tracking cameras (CAM_1,2_) to maintain alignment of the classical and quantum signals. A continuously variable ND wheel (ND_variable_) is used to dynamically change the channel loss as a function of time and emulate the overpass. Classical data are transmitted by two SFP transceivers, including the sifting traffic, EC, and PA. The transmit/receive optical signals for the classical lasercomms are combined to the same beamline via a BS. Secure keys are transmitted to a local computer (COM) and are stored locally to each subsystem (QTx, QRx).

There are three main subsystems that constitute the free-space QKD system: the QTx, QRx, and beam delivery terminals (BDTs). The QTx comprises a polarization encoder, operating at 843.9 nm, that encodes single photons according to the QKD protocol and a QRNG that provides random bits to the polarization encoder at 1 Gbps in real time. Both the polarization encoder and QRNG use a field programmable gate array (FPGA) to generate and process the data required to encode the photons. The QRx contains a polarization decoder connected to four single-photon avalanche detectors (SPADs). We use commercially available silicon SPADs due to their high detection efficiency and low dark counts. Last, the BDTs, which incorporate the PAT functionality, serve to multiplex and demultiplex the quantum, classical and beacon beams at the transmitter and receiver stations. Figure 2 shows a schematic of the experimental setup, including the QTx, QRx, and BDTs.

**Fig. 3.**
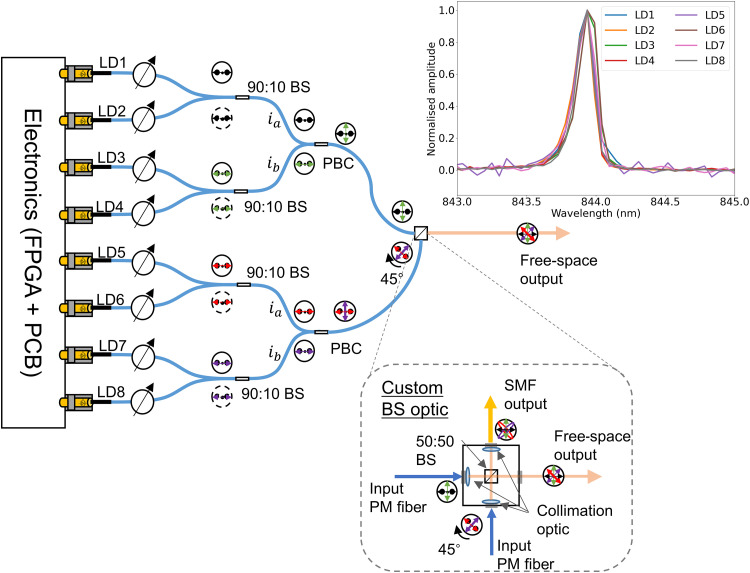
QTx polarization encoder. Schematic of the polarization encoder used to encode T12 protocol QKD to free space. Eight PM-coupled VCSELs are driven by an FPGA and custom PCB providing the fast signals to pulse the lasers according to the protocol. The lasers’ output power is set via an electronically controllable VOA and subsequently coupled pairwise via unbalanced fiber BSs (90:10 BS) to form signal and decoy intensity level light pulses. The outputs of the fiber BSs are coupled pairwise to polarization beam combiners such that the output has light pulses propagating along the slow and fast axes of the PM fiber. A custom nonpolarizing BS with two PM fiber inputs, one of which is rotated by 45° with respect to the BS axis, and two outputs, one directly to free space and another to single-mode fiber. Inset: Spectrum of each laser diode when filtered via two 0.2-nm free-space optical band-pass filters.

We consider the T12 protocol (*24*) for the satellite-to-ground QKD system, sending weak coherent optical pulses at a clock rate of 1 GHz, encoded in the polarization degree of freedom using eight gain-switched laser sources. Vertical cavity surface emission lasers (VCSELs) are used due to their low power consumption, high polarization extinction ratio, and large modulation bandwidth. The VCSELs are combined by an optical polarization combiner module to produce four polarization states at two intensity levels, which are required by the QKD protocol. A schematic of the eight-laser polarization combiner is given in Fig. 3. The inset of Fig. 3 shows the wavelength of the laser diodes after spectral filtering. Further details of the optical module can be found in Materials and Methods.

To ensure the security of a QKD device, there must be a source of provably random bits {0,1}, which are used to form the quantum key. A common source of theoretically secure random entropy is a QRNG (*30*), ideally producing at least one 133 bit of random entropy per clock cycle. These bits are used to randomly encode the photons carrying the quantum key. For gigahertz QKD systems, this means producing gigabits per second (Gbps) of random data; however, devices fulfilling these criteria tend to have high power consumption. In the case of satellite-based QKD, we would like to achieve high-bit rates while simultaneously keeping the power consumption as low as possible. To this end, we have developed a QRNG printed circuit board (PCB) that uses low power consumption integrated circuits, eschewing power-hungry electronic components such as high-bit rate analog-to-digital converters or RF amplifiers. The QRNG PCB is based on phase diffusion in gain-switched distributed feedback (DFB) lasers (*31*) input to an asymmetric interferometer (*32*) and sampling the output with a clocked comparator to produce a random bit stream at 1 Gbps. The random bit stream is received by the polarization encoder FPGA and expanded by a factor of 13 using a pseudo-random binary sequence (PRBS) module. We seed the PRBS in real time using the feed from the QRNG and encode the photons at the polarization encoder by randomly selecting the basis, bit, and intensity level (4 bits per clock cycle) as per (*33*).

The QRx implements a passive optical setup to decode the quantum states (see Fig. 2). The quantum signal is input to a triplet of birefringent wave plates to align the transmitter and receiver; specifically, two quarter–wave plates (λ/4) are used to align the reference frame of the receiver to that of the transmitter and a half–wave plate (*Q*), which is set at 0° and rotated about the fast axis to correct for any dephasing between orthogonal polarization components. The decoding basis selection is then passively made via a 90:10 nonpolarizing beam splitter (BS). The two orthogonal qubits are measured by a polarizing BS (PBS) and detected by single-mode fiber-coupled single-photon detectors. The detectors are connected to an FPGA for real-time time-tagging and sifting of the measured photon events. A custom PCB is used to condition the detector output ready to be sampled by the FPGA.

In our experiments, we also implement a PAT system using FSMs (FSM_1_ and FSM_4_), complementary metal-oxide semiconductor (CMOS) cameras (CAM_1_ and CAM_2_), and tracking beacons (B_1_ and B_2_). The PAT system has a field of view of 2 mrad, a pointing accuracy of better than 20 μrad, and a tracking latency of 5 ms. To emulate the coarse pointing error of the satellite toward the ground and the ground station toward the satellite, we use two FSMs (FSM_2_ and FSM_3_). The angle of the misalignment mirrors is randomly chosen every 50 ms via a pseudo-random number generator. The maximum misalignment that our system was able to tolerate while maintaining the classical communication links was 250 μrad. This matches the coarse pointing accuracy of state-of-the-art satellite and ground station platforms (*34*, *35*) and is greater than the expected pointing misalignment due to first-order turbulence effects (*36*).

The quantum, classical, and beacon signals were multiplexed/demultiplexed at the transmitter and receiver by a multiband BDT. The BDTs (BDT_1_ and BDT_2_) consist of a series of dichroic mirrors (DMs) to combine and separate each component signal at their respective wavelengths. These can be seen in Fig. 2, and further details can be found in Materials and Methods. The combined beams are passed through the FSMs for misalignment and tracking and then to an electronically controllable continuously variable neutral density (ND) wheel. The beam passed through a small portion of the ND wheel such that the multispectrum beam experienced an approximately uniform loss. We calibrated the ND wheel using a power meter to find the loss as a function of the wheel’s angle. This calibration was then used in conjunction with the estimated pass loss to dynamically introduce the channel loss to all of the optical channels simultaneously.

Synchronization and sifting were performed directly by the FPGAs of the QTx and QRx, via 1530-nm 1G small form-factor pluggables (SFPs), whereby the raw key material was generated according to the T12 protocol (see Materials and Methods). Completed sifted keys were then sent, by both the QTx and QRx, to their local computer via a 10G SFP link. In the sifting process, 1 megabit (Mbit) of sifted key material was generated before being saved to the local disk. The EC and PA processes were then performed on these 1-Mbit sifted key packets. While EC is always performed on a block containing 1 Mbit of raw sifted key, the number of 1-Mbit blocks that is used for PA was changed to explore how it affects the final amount of secret key. Communication relevant to the EC and PA processes was sent via 1G 1550-nm SFPs from the local computers.

## RESULTS

**Fig. 4.**
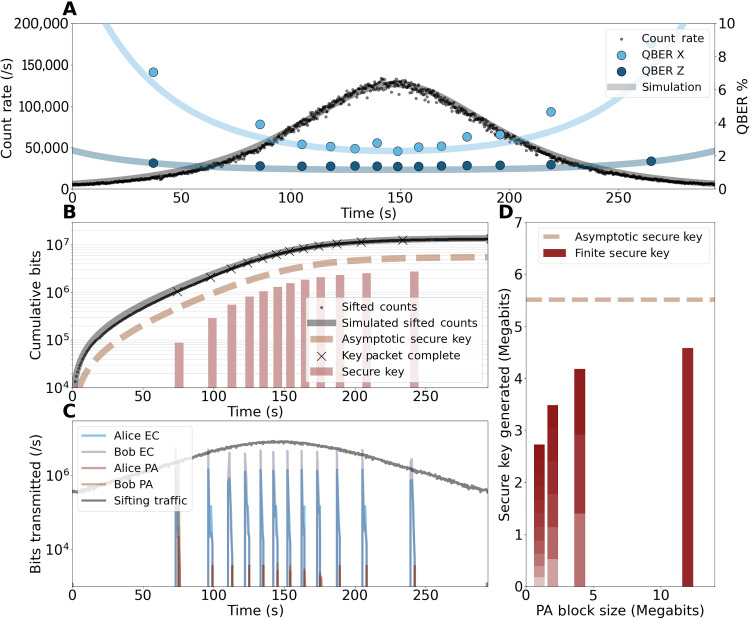
Emulation of optimal overpass. (**A**) Total count rate measured at the four QRx detectors as a function of the emulated satellite overpass time *t*_pass_. The measured data are plot alongside the simulated count rate using the total loss from Fig. 1B. The QBER, averaged over a key packet (1 Mbit) is plot (circles) along with the expected QBER from a model that uses the measured system parameters. (**B**) The cumulative measured counts for the emulated overpass are plot showing that a total of >1.3 × 10^7^ counts are retrieved in the overpass window. The cumulative asymptotic key can be compared (dashed line) to the cumulative distilled key (bars) when the block size of the PA is set to 1 Mbit = 1 key packet. (**C**) Traffic on the classical communications channel is shown for the sifting process (gray) and the EC and PA processes in each direction. A maximum data rate of close to 10^7^ bits is recorded. (**D**) Plot showing the effect of increasing the block size input to the PA. As the block size is increased, the total amount of secure key produced approaches the asymptotic limit (dashed line). The color gradient shows the additional key generated after each PA block has been completed, where the total key is made up of multiple blocks (if block size <12 Mbit). For a block size of 12 Mbit, the total secure key accumulated is 83% of the asymptotic key.

The QKD system was operated over a 3-m free-space link within our laboratory. See Materials and Methods for the full description of the experimental procedure. Figure 4A shows the count rate throughout the ∼294-s overpass alongside the simulated count rate from the channel loss predicted in Fig. 1B. We also plot the measured quantum bit error rate (QBER) for both the majority (*Z*) and minority (*X*) bases, alongside the expected variation in the QBER using a model that takes into account the system parameters (see Materials and Methods). The QBER is calculated after 1 Mbit = 1,048,576 majority photons have been sifted, which we refer to as a key packet. The values are plot at time *t*_packet_/2 (where *t*_packet_ is the time at which 1 Mbit of key has been successfully sifted), indicating the QBER for the key packet. We find a baseline QBER of 1.1%, which is limited by the detector jitter as discussed in the Supplementary Materials. The background counts were measured to be 130 counts/s summed across all four detectors. This is approximately the dark counts of the detectors; emulated overpasses with higher background counts can be found in the Supplementary Materials.

Figure 4B shows the accumulated sifted counts (qubits) over the course of the emulated overpass. We find that over 1.3 × 10^7^ counts are recorded by the end of the overpass. We also plot the accumulated asymptotic secure key. This provides an upper bound on the amount of secure key that can be generated by our system within this type of overpass. Following the successful sifting of a key packet, marked by × in Fig. 4B, the EC and PA software algorithms start and produce error-free quantum keys that are provably secure, with security parameter ε = 10^−10^. The total amount of secure key that has been produced throughout the overpass is displayed as bars within Fig. 4B. For the example shown here, the PA algorithm is applied to each error corrected key (block size = 1 Mbit = 1 key packet). Twelve sifted key packets are accumulated, from which we extract 2.72 Mbit of secure key after EC and PA. The final ∼528 kbit of sifted key are discarded as they do not fill a complete key packet. Figure 4C shows the traffic from the classical channels over the course of the emulated overpass for the case of a PA block size of 1 Mbit. The average sifting traffic from the 1G 1530-nm SFP is shown and reflects the total counts detected. For each detected photon, 128 bits of classical traffic are exchanged, 64 bits in each direction. These data contain information about the photon time of arrival (QTx and QRx), the measurement basis choice (QRx), sending basis (QTx), sending flux (QTx), and the key packet that the photon belongs to (QTx and QRx). For the events recorded at the minority detectors, the bit sent by the QTx is announced to provide an estimation of the errors. Once the sifted key packet has been transferred to the computer, the EC and PA algorithms start to run, and we see that the traffic on the 1550-nm 1G SFP spikes to ∼6 Mbps. The data are mostly exchanged in the EC process, while PA requires only a random hashing seed to be shared. For full details on these processes, see Materials and Methods. Last, in Fig. 4D, we show the effect of increasing the block size input to the PA from 1 key packet up to a maximum of 12 key packets. We see that as the PA block size is increased, we approach the asymptotic key limit, reaching a maximum of 4.58 Mbit. Therefore, we achieve a ratio of the final output key to the total amount of sifted key material of approximately 1 of 3. The finite key accumulated for this block size reached 83% of the asymptotic limit.

**Fig. 5.**
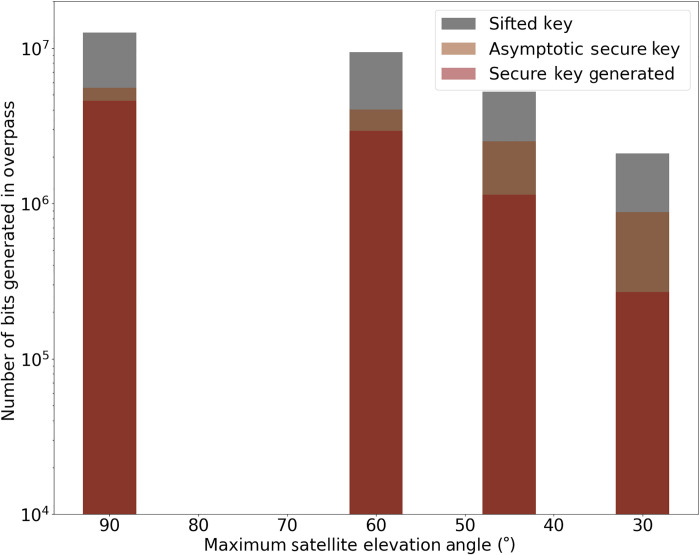
Key generation for different satellite passes. The total sifted key, asymptotic key, and finite secure key are plot as a function of the maximum elevation angle for four different emulated satellite trajectories. The trajectories have a maximum elevation to the ground site of 90°, 60°, 45°, and 30°. In each case, we compute the key for the maximum block size possible, which was 12, 9, 5, and 2, respectively.

The overpass explored hitherto, with an average channel loss of 34.00 dB, shows that we are able to approach the asymptotic limit for secure key length when large PA block sizes are considered. We have also explored trajectories with higher average channel loss and found that our system can sustain all of the communication channels and provide a positive quantum key with satellite maximum elevation as low as 30°, corresponding to an average loss of 38.54 dB. In Fig. 5, we show the amount of key generated per overpass, the asymptotic key expected for the overpass, and the secure key generated by our system for satellite trajectories with 90°, 60°, 45°, and 30° maximum elevation. This results in an average channel loss for each overpass of 34.00, 34.82, 36.03, and 38.54 dB, respectively. The start (stop) elevation was fixed at 20° (160°), resulting in pass durations of 294, 286, 266, and 210 s, respectively. We note that in certain circumstances, where intermittent communication with the satellite may occur (for instance, due to weather conditions), it may be preferable to operate the system with small PA block sizes. We have shown that our system is still capable of producing large quantum keys even for low maximum elevation angle passes.

## DISCUSSION

While our system is capable of emulating the losses expected for a satellite overpass, the ability to accurately share an absolute time reference between the transmitter and receiver is challenging to recreate within a laboratory setting due to the varying link distance as a function of time. Nonetheless we can estimate the photon buffer required to accommodate the link distances expected. The photon buffer is used to store detected events ready for reconciliation via the sifting process. The depth of this buffer should be at least twice the maximum trip time between the satellite and ground. For the optimal overpass and starting at a link distance of 1200 km, the transit time of light is 4 ms, suggesting that a minimum buffer depth of 32 Mbit is required for a QKD clock rate of 1 GHz. For our laboratory tests, we implement an 8-Mbit photon buffer, with the entire FPGA implemented design using 25.58% of the block random-access memory (BRAM) resource. This suggests that increasing the buffer to 32 Mbit would require a larger FPGA size or external RAM. This is within the resource of modern FPGA chipsets, which can have >75 Mb of BRAM. We also note that Doppler effects would affect the perceived clock rate of the communication channels at the ground site. One of the advantages of our system is that the classical traffic experiences the same optical channel as the quantum and will therefore compensate for the temporal drift expected. One issue that could lead to a bottleneck in communication is in the EC and PA algorithms, which require multiple rounds of communication. To confirm that the EC and PA communication is able to operate with the latency expected, we add 4 ms of latency at each SFP interface (1550 nm); with this latency added, we see a relatively small increase (⁓1 s) in the time it takes to complete the EC and PA processes. All data shown in the manuscript are with this fixed latency added to the 1G SFP interface. Alignment of the absolute time has been addressed by many studies, and a number of solutions have been proposed, including using synchronization beacons, global positioning systems, de Bruijn sequences, etc. (*37*–*39*). However, the implementation of this synchronization is outside the scope of this work and will be the focus of future research.

By using a 1-GHz clock rate, we have demonstrated that it is possible to achieve an order-of-magnitude increase in the size of the secure key output for a single overpass (*18*) even when relatively small telescope apertures are considered. Furthermore, all of the classical data relevant to the quantum key are transmitted within the overpass, allowing for the final quantum key to be distilled in real time. This means that information relevant to the quantum key does not need to be stored at the satellite, which can approach 10^12^ bits in just a few hundred seconds. We believe that our system demonstrates that real-time SatQKD is close to becoming a commercially realizable technology that will be able to service large QKD networks and connect distant parties to communicate securely. Furthermore, by saving resource aboard the satellite, smaller platforms can be used that are more cost effective and can therefore be deployed in large constellations, servicing a global QKD network.

## MATERIALS AND METHODS

### Optical polarization combiner

The optical polarization combiner module (see Fig. 3) multiplexes eight VCSEL lasers to produce the polarization states at the desired flux for the QKD protocol. First, the lasers are coupled to polarization maintaining (PM) fiber in which the light pulses travel along the slow axis. They are then coupled in signal-decoy pairs using unbalanced (90:10) fiber BSs. The average photon flux μ is set using variable optical attenuators (VOAs), which are individually controlled by an eight-channel DC voltage regulator. We set the four signal lasers to an average photon flux of μ*_u_* and the other four decoy lasers to μ*_v_* = μ*_u_*/6 by fine adjustment of the VOAs; vacuum states are encoded by not pulsing any of the lasers. After this step, we have four fiber outputs each with light pulses of two intensity levels that are propagating along the slow axes of the PM fiber. Orthogonal polarization states are generated by combining the output of two BSs using a polarization beam combiner. A polarization beam combiner couples light at the two input ports, *i_A_* and *i_B_*, to the slow and fast axes of the output port respectively. Thus, the output of the polarization beam combiner contains laser pulses in orthogonal polarization states. Last, these outputs are combined at custom BS optic. This custom BS has two input PM fibers, with one of the inputs rotated by 45° with respect to the BS axis, to generate the mutually unbiased polarization states, ∣*D*⟩ and ∣*A*⟩. A free-space BS combines the ∣*H*⟩ and ∣*V*⟩ states from the unrotated input and ∣*D*⟩ and ∣*A*⟩ pulses from the rotated input and couples the output either directly to free-space or alternatively to a short length of single-mode fiber via a collimation optic. The advantage of coupling directly to free space is that thermal transients will not affect the polarization states generated as the states only propagate in PM fiber. Coupling to single-mode fiber provides protection against possible side-channel attacks whereby the two basis states combined at the output BS are coupled at a slight angle, i.e., noncollinearly (*40*). In our experiments, we use the free-space output and find that no feedback mechanism is required to keep the transmitted polarization states aligned to the QRx.

The lasers are soldered to a custom PCB, which supplies the DC bias and the fast RF signal via a high-bandwidth laser driver. An FPGA is used to encode the laser pulses at a clock rate of 1 GHz. The lasers operate in gain-switched mode ensuring that each laser pulse has a random phase, which ensures the security of the protocol. Each laser diode is temperature controlled using a thermoelectric cooler (TEC) to tune the emission wavelength. The TEC is capable of stabilizing the temperature over a range of >35° with a precision of ±0.01°, providing a wavelength tuning range of approximately 2.5 nm. We set all of the lasers to a central wavelength of 843.9 nm. A pair of free-space optical band-pass filters (*F_Q_*), with a full width at half maximum of 0.2 nm, is used at the output of the QTx to ensure that spectrum of each laser is indistinguishable.

### BDTs with fine PAT system

De/multiplexing of the broadband optical signals is performed by a set of long- and short-pass DMs. The first of the DMs allows the incoming beacon to pass to the tracking camera and allows the outgoing beacon to be coupled to the main beam line. At the transmitter, a long-pass dichroic (DM_*B*1_) with a cut-on wavelength of 650 nm was used, while at the receiver, we used a short-pass dichroic (DM_*B*1_) with a cut-off wavelength of 650 nm. The outgoing beacons were multiplexed/demultiplexed with the quantum signal via a short-pass DM [DM_*Q*1(2)_], with a cut-off wavelength of 800 nm, reflecting the quantum signal into (out of) the QTx (receiver). Last, the bidirectional classical communication traffic for sifting, EC and PA were multiplexed/demultiplexed to/from the beam line via a short-pass DM [DM_*C*1(2)_] with a cut-off wavelength of 1200 nm.

The beacons B_1_ and B_2_ use DFB lasers with central wavelengths of 630 and 650 nm, respectively. The quantum source has a central wavelength of 843.9 nm. Two separate classical communication channels were used, the sifting traffic via a 1G 1530-nm SFP operating at a line rate of 1 Gbps, and the EC and PA via a 1G SFP at 1550 nm. The classical channels were combined via a coarse wave division multiplexer, whereby the two channels were on adjacent bands. The transmitted and received signals were separated via a standard C-band free-space BS. The fine point-and-track (PAT) subsystem incorporated into the multi-band BDTs use FSM and a CMOS camera. The field of view of the fine PAT system is ±2 mrad, while the FSM1,2 and FSM3,4 have a mechanical steering range of ±20 and ±3 mrad, respectively. The CMOS cameras (CAM_1,2_) have a full frame size of 1280 × 1024 pixels and a frame rate of 165 Hz at full frame size. The PAT subsystem uses only 336 × 256 pixels and runs the cameras at 900 Hz. The overall latency of the PAT system is measured to be <5 ms.

### Transmission efficiency

The total transmission efficiency of a satellite-to-ground QKD link can be estimated as a function of the satellite elevation angle (α), full-angle beam divergence (θ), PAT error (ε_pat_), and the wavelength (λ), as given in Eq. 1 (*41*). This considers the impact due to diffraction (η_diff_), atmospheric transmissivity (η_atm_), PAT efficiency (η_pat_), coupling efficiency to single-mode fiber (η_coupling_), photon detection efficiency (η_pde_), the efficiency of the optics at the receiver (η_rx_), and decoherence of the beam via turbulence effects (η_turb_).

$$\eta (\alpha ,\theta ,\lambda )=\eta_{\mathrm{diff}}(\alpha ,\theta )\eta_{\mathrm{atm}}(\alpha )\eta_{\mathrm{turb}}\eta_{\mathrm{pde}}\eta_{\mathrm{rx}}\eta_{\mathrm{coupling}}\eta_{\mathrm{pat}}$$ (1)

$$\eta_{\mathrm{diff}}(\alpha ,\theta )=\frac{D_{r}^{2}\eta_{\mathrm{obs}}^{2}}{{\left[\theta L(\alpha )\right]}^{2}}$$ (2)

$$\eta_{\mathrm{atm}}=\eta_{\lambda }^{\csc(\alpha )}$$ (3)

The channel efficiency, which is implemented with an ND wheel and fixed ND filters in the emulator, is given by η_chan_ = η_diff_(α, θ)η_atm_(α)η_turb_. The remaining terms in Eq. 1, namely, η_pde_, η_rx_, η_coupling_, and η_pat_, are intrinsic to the experimental setup. The APD detection efficiency is denoted η_pde_ and losses at the optics used in the BDT and polarization decoder are denoted by η_rx_. The coupling efficiency to single-mode fiber η_coupling_ was 0.56. We note that turbulence effects will affect the insertion loss of our system. First-order turbulence effects, also known as “seeing,” degrade the pointing stability of the beam; this effect, alongside the pointing stability of the satellite and telescope platforms, is emulated by FSMs at each BDT in our experiments. The pointing error induced by additional FSMs (FSM2 and FSM3 in Fig. 4) is tracked and corrected by the PAT system within each BDT. Loss due to the pointing error of the tracking system is 0.4 dB, which provides the transmission efficiency for PAT system η_pat_ = 0.912. Higher-order turbulence effects, which cause perturbation to the wavefront (also known as scintillation), cause decoherence of the beam and are not corrected by the FSMs. Instead, this requires higher-order correction, for instance, via adaptive optics (*42*). Therefore, we expect an additional loss, which varies depending on the precise details of the atmospheric turbulence experienced, i.e., site location, weather, time of day, etc. Here, we chose an optimistic yet typical value of η_turb_ = 0.631 (2-dB loss), which has been found through experimental measurement in (*36*) to vary between 0.09 and 4.3 dB. This can be considered to effectively reduce the coupling efficiency to 35% for our experiments. Assuming nighttime operation, this value is realistic to achieve through the use of modern (multi-order) adaptive optics systems (*43*). By combining η_turb_, η_rx_, η_coupling_, η_pat_, and η_pde_, we find the total loss at the QRx to be 8.5 dB (the measured transmission efficiencies are shown in Table 1). We believe that this can be improved by optimizing the losses through the optics in the receiver, i.e., increasing η_rx_ or by improving the coupling efficiency to single-mode fiber. Furthermore, the coupling efficiency due to turbulence effects will change as a function of the elevation angle, which is not accounted for in this study as we do not expect a large variation for nighttime operation; instead, we have assumed a pessimistic value (expected for low satellite elevations) across the entire pass. However, this variation should be taken into account when daytime operation is under consideration (*43*).

**Table 1.** Transmission efficiencies for the optical channel model.

Parameter	Transmission efficiency	Loss (dB)
Turbulence η_turb_	0.631	2
Receiver optics η_rx_	0.753	1.23
Photon detection efficiency η_pde_	0.58	2.36
SMF coupling η_coupling_	0.56	2.51
Pointing error η_pat_	0.912	0.4

The diffraction efficiency in the far-field, η_diff_, is given by Eq. 2 (*44*), where *D*_r_ is the diameter of the receiver telescope, η_obs_ is the transmissivity of the receiver telescope due to the central obstruction (0.91, for 30% diameter obstruction), and *L* is the link distance between the satellite and the optical ground receiver, which is given as Eq. 4. The parameter η_atm_ is given by Eq. 3, where η_λ_ is the atmospheric transmissivity at zenith (*45*) and changes as a function of wavelength.

$$L(\alpha )=-r_{E}\cos\left(\frac{\pi }{2}-\alpha \right)+\sqrt{r_{E}^{2}\cos{\left(\frac{\pi }{2}-\alpha \right)}^{2}+h_{\mathrm{orbit}}^{2}+2h_{\mathrm{orbit}}r_{E}}$$ (4)

The total time that the satellite is visible is given by Eq. 5 (*46*). This equation defines the total pass duration (τ), based on the minimum and maximum satellite elevation angles (α_min_ and α_max_), the orbit inclination (ϕ), the angular velocity of the satellite (ω_S_), the angular velocity of Earth’s rotation (ω_E_), the radius of Earth (*r*_E_), and the altitude of the satellite orbit (*h*_orbit_). By setting α_min_ = α, we can relate each satellite elevation angle to a time, *t*_pass_(α), within the visibility window τ, through Eq. 5 and then use Eqs. 1, 2, 3, and 4 to calculate the total transmission efficiency at each time during the satellite overpass. The experiments consider a satellite-to-ground link setup with parameters shown in Table 2. Plots for the estimated channel losses are shown in the Supplementary Materials.

$$\tau \approx \frac{2}{\omega_{S}-\omega_{E}\cos(\phi )}\cos^{-1}\left(\frac{\cos\{\cos^{-1}\left[\frac{r_{E}}{r_{E}+h_{\mathrm{orbit}}}\cos(\alpha_{\min})\right]-\alpha_{\min}\}}{\cos\{\cos^{-1}\left[\frac{r_{E}}{r_{E}+h_{\mathrm{orbit}}}\cos(\alpha_{\max})\right]-\alpha_{\max}\}}\right)$$ (5)

**Table 2.** Satellite-to-ground QKD link parameters.

Parameter	Value
Orbit inclination ϕ (°) and altitude *h*_orbit_ (km)	90 and 500
Orbits with maximum satellite elevation α_max_ (°)	90°, 60°, 45°, 30°
QKD full beam divergence θ (μrad)	17.2
Atmospheric transmissivity (η_λ_) at 843.9 nm	0.5
PAT system accuracy ε_pat_ (μrad)	3
Transmitter telescope aperture (diameter) (m)	0.1
Receiver telescope aperture (diameter) (m)	0.6
Receiver telescope obstruction (%)	30
QKD source wavelength (nm)	843.9

### T12 QKD protocol

We implement the T12 protocol (*24*), which is an optimized version of the decoy-state BB84 protocol. We select four equatorial states of the Poincare sphere, corresponding to the polarization states ∣*H*〉, ∣*V*〉, ∣*A*〉, and ∣*D*〉, and into which the information relative to two bases {*Z*, *X*} and two bits {0,1} is encoded. Optimally biasing the basis selection probabilities *p*(*Z*) > *p*(*X*) ensures a maximum number of events where the sending and measurement bases match while guaranteeing a sufficient number of events in the minority basis to provide a reliable estimation of the error, δ.

The protocol requires that the average photon number per encoded light pulse (symbol) is selected at random from three intensity levels, which, by convention are referred to as signal (*u*), decoy (*v*), and vacuum (*w*). We send the bases with probabilities of *p_Z_* = 15/16 and *p_X_* = 1 − *p_Z_*. The relative intensity levels of the three states are set to μ*_u_* = 0.405 photons per symbol, μ*_v_* = μ*_u_*/6, and μ*_w_* < μ*_u_*/500, which we found to be optimum for our system. Following (*24*), we encode the bit, basis, and intensity variables, which we denote *Q*, *B*, and *I*, respectively, where *Q* ≡ {0,1}, *B* ≡ {*X*, *Z*} and *I* ≡ {vacuum, signal, decoy}, with probabilities *p*(*Q* = 0,1) = 1/2, *p*(*B* = *X*) = 1/16, and *p*(*I* = vacuum) = 1/16, *p*(*I* = decoy) = 15/256.

### QBER and asymptotic secure key model

We estimate the asymptotic key in our pass according to the security proof of the T12 protocol from (*24*). The model uses the measured system parameters and count rates from our experiments, without any fitting parameters. This includes the detector efficiency, photon flux of the three intensity states, the relative probability with which the quantum states are sent, and the base qubit error rate. In the absence of imperfections, after EC and PA, the asymptotic secret key generation rate is given by *R* = 1 − *H*(*e*) − *H*(δ), where *H*(*x*) = −*x*log_2_(*x*) − (1 − *x*) log2(1 − *x*) is the Shannon binary entropy.

### Running the emulated pass

The beacon, quantum, and classical signals were collimated to a beam waist of 1.3, 4, and 4.65 mm, respectively, and then sent through the BDTs. We prepare to start an emulated overpass by ensuring that the QTx was operating at the correct flux for the protocol. The misalignment FSMs (2 and 3) are started such that a random pointing error of between 0 and 250 μrad is induced in each direction. Then, we begin the tracking so that the tracking FSMs (1 and 4) couple the maximum power to the receiving detectors for the classical and quantum channels. The ND wheel is then set to the minimum count rate for the overpass. Our control software for the QTx allows us to stall the sifting process such that key material is not saved to the local buffer. Once we are ready to start an overpass, an automated routine to move the ND wheel begins, and we unstall the sifting process at the QTx. As the ND wheel is rotated and the channel loss starts to decrease, we see that the count rate measured at the QRx increases as expected from the model. Once the overpass has finished, the QTx is stalled again such that no more events are stored.

### Error correction and privacy amplification

The FPGA-based sifting process outputs raw sifted key packets at the two user locations. These sifted keys are not identical because of nonzero QBER. Postprocessing of the keys is therefore required for EC. This is achieved through communication between the users over a public (authenticated) channel. This process unfortunately leaks some information about the keys to an eavesdropper. Therefore, a further postprocessing stage of PA is required for randomness extraction, to “process out” any knowledge an eavesdropper could have about the key, taking into account statistical fluctuations of finite-sized samples. This reduces the key length but results in provably secure QKD keys with a bounded security parameter.

For EC, we use the widely used QKD cascade algorithm (*47*). Cascade is a simple yet highly efficient protocol, which involves interactive communication. This splits the key packets into blocks then performs a binary search over multiple rounds to find and correct errors. Other EC algorithms could be used, such as low-density parity check codes. These have the advantage of requiring one-way communication, better suited for high latency links, although they are more complex and require careful optimization for a given link. For links with a fast-varying QBER (e.g., varying from <2% to >8%, Fig. 1), Cascade offers improved efficiency over a wider range of link parameters. For PA, we apply the T12 security proof (*24*) to compute the required compression ratio given the measured link parameters, then apply randomness extraction (using an optimized number-theoretic transform–based hashing algorithm) to compress the error-corrected key packet to a reduced size, forming the provably secure output keys.

In our experiments, we add 4 ms of latency at each node, which is the maximum expected for the overpass presented. This indicates that the EC and PA protocols chosen are suitable candidates for long distance operation.

## Supplementary Materials

### This PDF file includes:

Supplementary Text Figs. S1 to S8 Table S1 References
