# Supplementary material for: Real-time gigahertz free-space quantum key distribution within an emulated satellite overpass
Source: Sci Adv. 2023 Dec 1;9(48):eadj5873. doi: 10.1126/sciadv.adj5873 (PMC10691775; doi:10.1126/sciadv.adj5873)
Supplement: Supplementary file 1 — Supplementary Text Figs. S1 to S8 Table S1 References [file sciadv.adj5873_sm.pdf]

Science Advances
AAAS

Supplementary Materials for

Real-time gigahertz free-space quantum key distribution within an emulated satellite overpass

Thomas Roger *et al.*

Corresponding author: Thomas Roger, thomas.roger@toshiba.eu

Sci. Adv. **9**, eadj5873 (2023)
DOI: 10.1126/sciadv.adj5873

This PDF file includes:

Supplementary Text
Figs. S1 to S8
Table S1
References

Supplementary Materials

Quantum transmitter output spectrum. To ensure the security of the QKD system the quantum transmitter should encode photons according to the protocol specified. Side channels, which may be exploited by an eavesdropper to compromise the security of the system can occur when the QKD protocol is not implemented correctly. For instance if the encoding state can be determined through measurement of another of the photon's properties, such as the wavelength. In figure S1 the emission spectrum of the 8 lasers used to produce the quantum signal are plot. The top panel shows the emission spectrum when the lasers are at room temperature i.e. the TEC is off. The middle panel when the TEC controllers set each of the central emission wavelength to 843.9 nm and the bottom panel after spectral filtering via a pair of 843.9 $\pm$ 0.2 nm bandpass filters. The central emission wavelength is red shifted at higher temperatures. To align the 8 lasers wavelength the temperature controllers are tuned between a minimum temperature of 15° and maximum temperature of 52°.

System operation. To achieve a full QKD session within a single overpass of the satellite, we implemented the sifting and EC & PA communication protocols via lasercomms. This increases the bandwidth for the communication channels compared to radio frequency (RF) communication, which is typically used to communicate with LEO satellites. We show that the classical data traffic can reach upwards of 7 Mbps for a single channel, which includes the overhead data for the communication protocols. This suggests that the interfaces could be operated at lower clock rates to achieve higher signal to noise ratio if required, or more data could be multiplexed onto the channels indicating that higher clock rates for the QKD system could be considered and would not face a bottleneck due to the sifting or EC & PA processes.

Channel loss in emulated overpass. In our emulation of the satellite overpass, the QKD link experiences the full expected channel loss. We induce a fixed 26.55 dB channel loss in the QKD link and the remaining dynamic loss of up to 13 dB is added by the ND wheel mounted on a rotation stage. The classical beams have a fixed loss of 6dB and additional dynamic loss of up to 13 dB by the same ND wheel. Therefore, the classical channels do not see the full expected channel loss of the overpass. This is due to the fact that we use a transmit power of $\sim$0 dBm for the classical links. The SFPs used in our experiment to transmit/receive the 1Gbit/s classical links have a power budget of 41 dB. This can be expanded to $>$65 dB by using optical amplification at the transmitter provided large aperture is used, and by using better sensitivity detectors. Such large power budget can allow implementation of full loss in the classical channel. Similarly the beacon beams currently see a dynamic loss of up to 13dB, which can be increased if the beacon laser is operated at high power.

Effect of background counts. Increasing the background counts leads to a larger quantum bit error rate due to a reduced signal to noise ratio. This is especially prevalent in the minority basis where there are fewer counts per second. In figure S2 the background counts are increased from

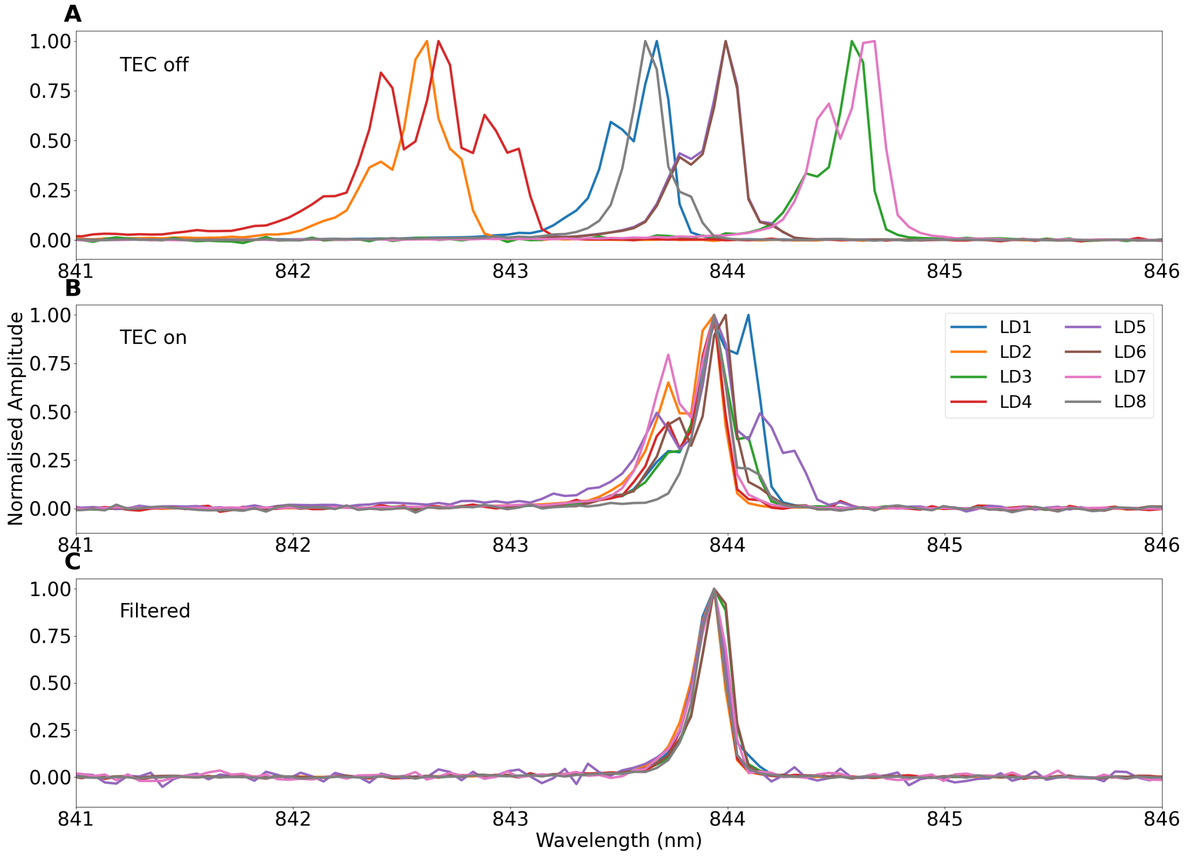

Figure S1: **Output spectrum of quantum transmitter.** Top panel (**A**): Optical spectrum of each of the 8 lasers used at the QTx polarization encoder without temperature control (TEC off). Middle panel (**B**): with TEC on and bottom panel (**C**): with two 843.9 $\pm$ 0.2 nm bandpass filters.

approximately the detector dark counts of 130 counts/s up to 6100 counts/s summed across all 4 detectors. The background counts were increased with a fixed ratio between majority and minority bases of 15/16:1/16. This approximates the effect of increased background flux that is coupled via the telescope of the receiver to the polarization decoder. We do not assume a particular model for the variation of the background counts as a function of the overpass and instead used a fixed value. The data was taken assuming the optimal case of a maximum elevation of the satellite of 90° and the secure key was totaled across the pass for an input size of 1 Mbit to the privacy amplification algorithm.

Auxiliary channel overpass loss. We also note that the loss experienced by each of the auxilary channels is different depending on whether the signals are sent from satellite-to-ground or ground-to-satellite. In figure S3 we plot the channel loss for the classical data signal and

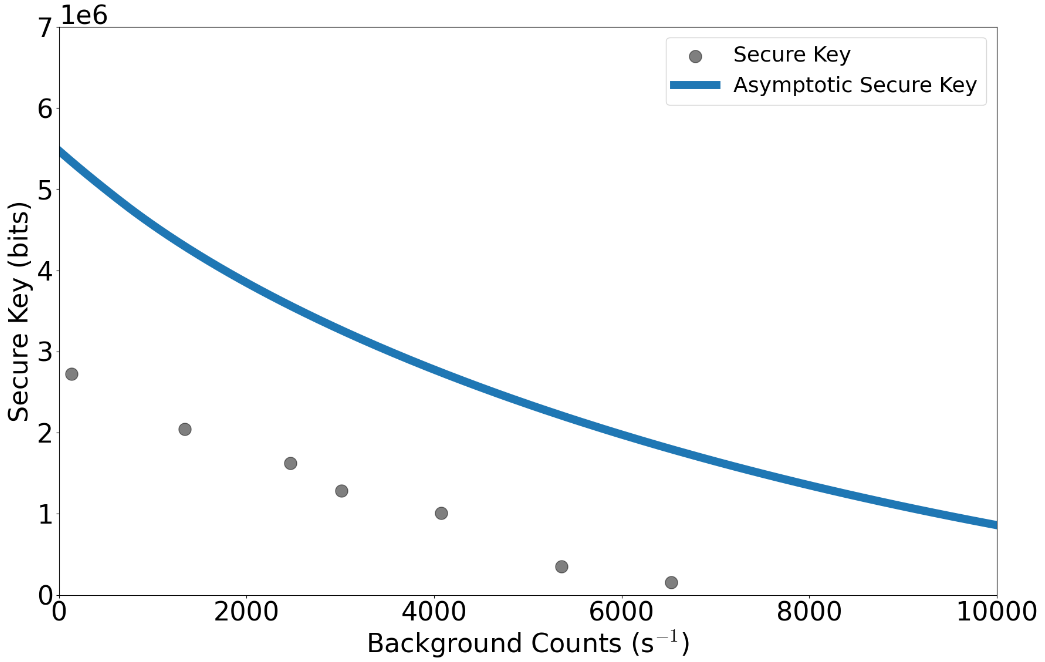

Figure S2: **Secure key generated in presence of increased noise.** Plot showing the variation of the asymptotic and measured secure key as a function of the background counts at the receiver detectors. The background counts were increased with a fixed ratio of 15/16:1/16 between the majority and minority detectors. Each measured value is the sum total of the secure key when a block size of 1 Mbit was used as the input to PA.

beacons for both the uplink (UL) and the downlink (DL) alongside the expected loss for the quantum channel, which is only DL due to the transmitter being on board the satellite. The DL channel losses have been calculated with the same model as used for the quantum channel but with updated parameters based on the emission wavelength, full-angle beam divergence and atmospheric loss at zenith [48]. These parameters are presented in table S1. For the UL, we used the channel model presented in [49] with HV 5-7 (sea level site) parameters, which takes the impact of atmospheric seeing into account. The classical uplink has a larger dynamic loss than experienced in our experiments but is still within the dynamic range of standard telecom SFPs, like the ones used within these experiments. To account for the additional dynamic range required we add 7 dB of loss to the uplink classical channel (1530 nm & 1550 nm). This demonstrates that the SFPs used within our experiments have the required dynamic range to perform communication and synchronization across the entire overpass.

Quantum receiver, passive basis choice. Ideally, a BS of splitting ratio 15/16:1/16 would be used or the transmitted encoding probability matched to the receiver, however in our system

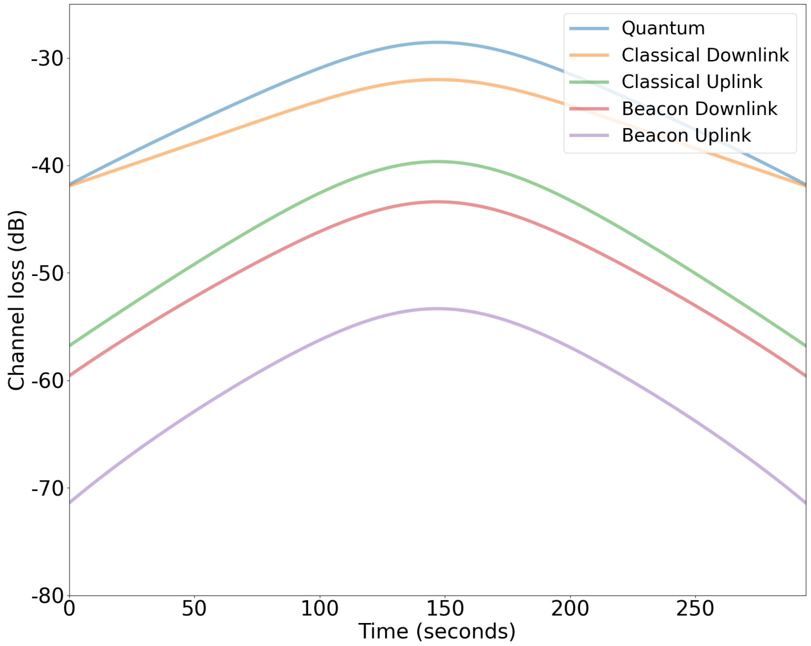

Figure S3: **Emulated optical channel losses.** Channel loss for the quantum, classical (uplink and downlink) and beacon (uplink and downlink) optical channels for the apertures assumed in our experiments. These losses assume a pass with maximum elevation of 90°.

Table S1: Auxiliary channel uplink and downlink parameters

**Parameter**	**Value**
θ_{1550DL}, θ_{1550UL}, θ_{B1}, θ_{B2}	31.2, 20, 100, 100
η_{1550DL}, η_{1550UL}, η_{B1}, η_{B2}	0.75, 0.75, 0.35, 0.33

this was not possible due to the implementation of the FPGA code at the transmitter and lack of availability for beamsplitters with the desired ratio. Here, we reduce the coupling efficiency for the minority detectors to provide the desired measurement basis selection probability according to the protocol. This does not affect the security of the protocol but slightly reduces the achievable key rate.

Data generated in a SatQKD overpass. We can estimate the total data that is generated in a typical overpass and would require storage if the sifting, EC & PA were to be performed by post-processing. Assuming an overpass of $\sim$294 seconds and the clock rate of QKD system used here (1 GHz), the total amount of data that should be stored by the transmitter is $\sim 1.2\times 10^{12}$ bits = 0.147 TB.

Detector jitter. The QBER of our system is limited in part due to the single photon detectors used at the QRx. These SPADs have an average jitter of 400 ps measured at the full width at half maximum (FWHM). However, they also exhibit a long tail persisting for longer than 1ns at greater than -20 dB (see fig. S4). This adds approximately 0.5-1 % to the baseline QBER. By reducing the clock rate to 500 MHz we see an improvement in the QBER, however do not expect an overall improvement in the secure key rate due to fewer qubits being sent per unit time. We estimate the contribution to the QBER at 1 GHz to be between 0.5-2 % depending on the specific detectors used, the fibers used to couple the light to the detectors and the count rate [50].

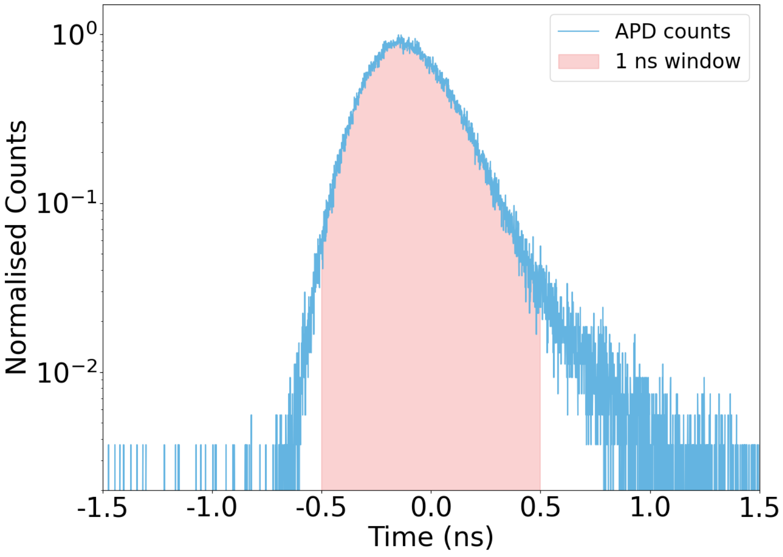

Figure S4: **Single photon detector jitter.** A typical histogram measurement of the counts measured at one of the single photon detectors for a count rate of 90 kc/s. We see that the counts persist for longer than 1 ns despite the optical pulse width of < 100 ps.

Single mode fiber. In our experiments we employ single mode fiber at the QRx and for the classical laser comms. In a real SatQKD link, turbulence effects would negatively impact the coupling efficiency. For the quantum signal this can be accommodated by using multimode fiber or by using adaptive optics at the ground station. Note that we already take into account diffraction effects for the received quantum signal in our model. If MMF is used at the QRx we expect an increase in coupling efficiency accompanied by an increase in QBER due to additional detector jitter [51], one of the causes of this effect is group delay dispersion (GDD), which can be reduced with the use of graded index MMF. We expect that these effects would roughly cancel each other out and will be studied in detail in the future. For the classical communications

single mode fiber is used in order to propagate the two optical channels through the CWDM. MMF may also be employed to receive the signals at the SFPs but would require that our BDTs are modified to include free-space (de)multiplexing of the classical signals via a dichroic mirror with a cut-off wavelength of around 1540 nm.

Maximum elevation. To study the effect of the satellite pass on the amount of secure key that can be produced we run our experiment for overpasses with different maximum elevation angle. In figure S6, S7 and S8 we show experimental data for maximum elevations of $60°$, $45°$ and $30°$. The time that the satellite is above $20°$ elevation for each of the passes is approximately 294, 286, 266 and 210 seconds respectively. We also plot the channel loss for each of the satellite passes in figure S5.

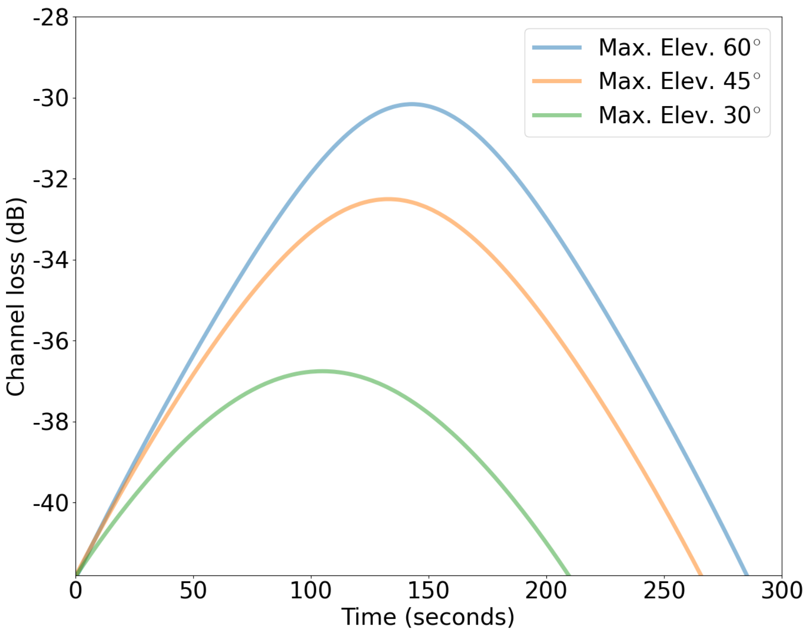

Figure S5: **Quantum optical channel loss for non-optimal satellite passes.** Channel loss for each satellite overpass studied with maximum elevation $60°$, $45°$ and $30°$.

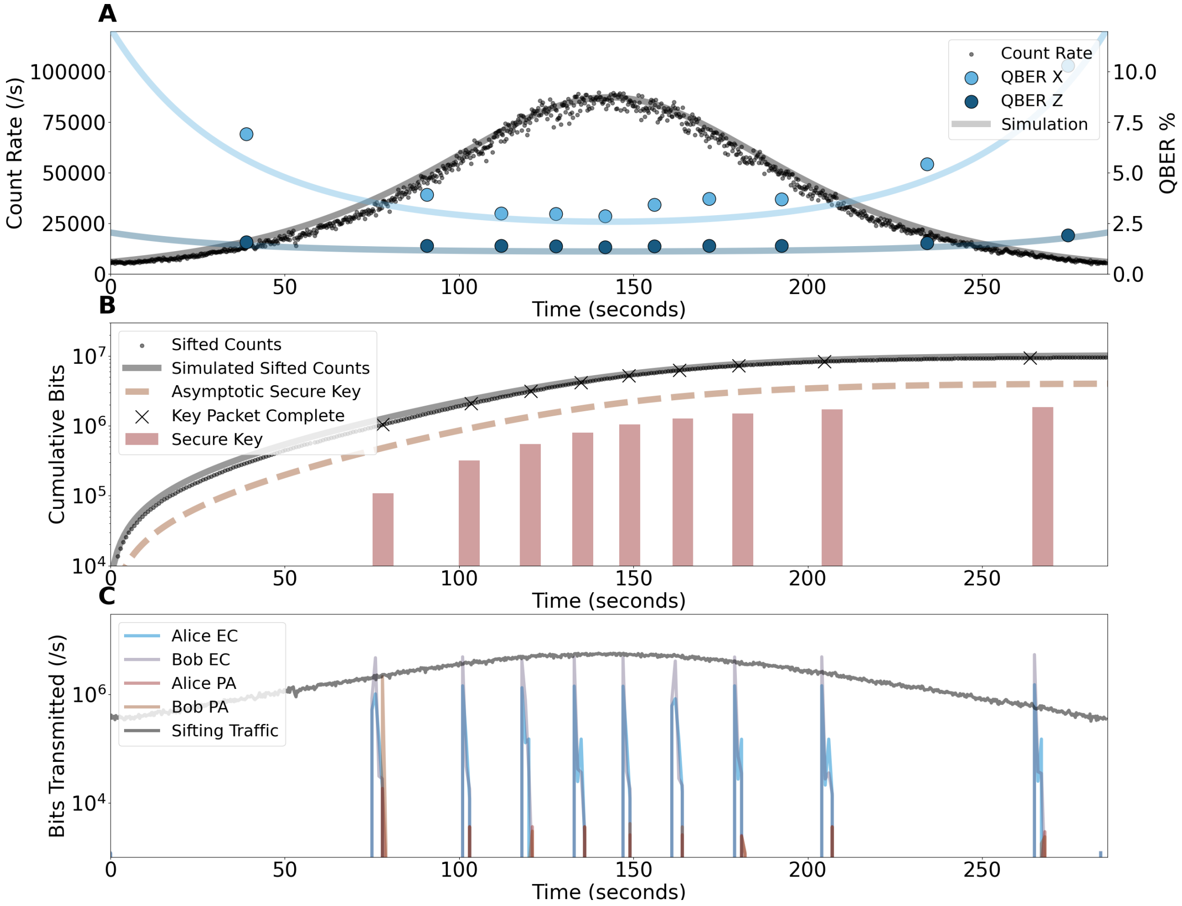

Figure S6: **60° maximum elevation overpass.** (**A**) Count rate measured at the 4 QRx detectors as a function of the emulated satellite overpass time t_{pass}. The measured data is plot alongside the simulated count rate. The QBER, averaged over a key packet (1 Mbit) is plot (circles) along with the expected QBER from a model which uses the measured system parameters. (**B**) The cumulative measured counts for the emulated overpass are plot. The cumulative asymptotic key can be compared (dashed line) to the cumulative distilled key (bars) when the block size of the PA is set to 1 Mbit = 1 key packet. (**C**) Traffic on the classical communications channel is shown for the sifting process (grey) and the EC & PA processes in each direction.

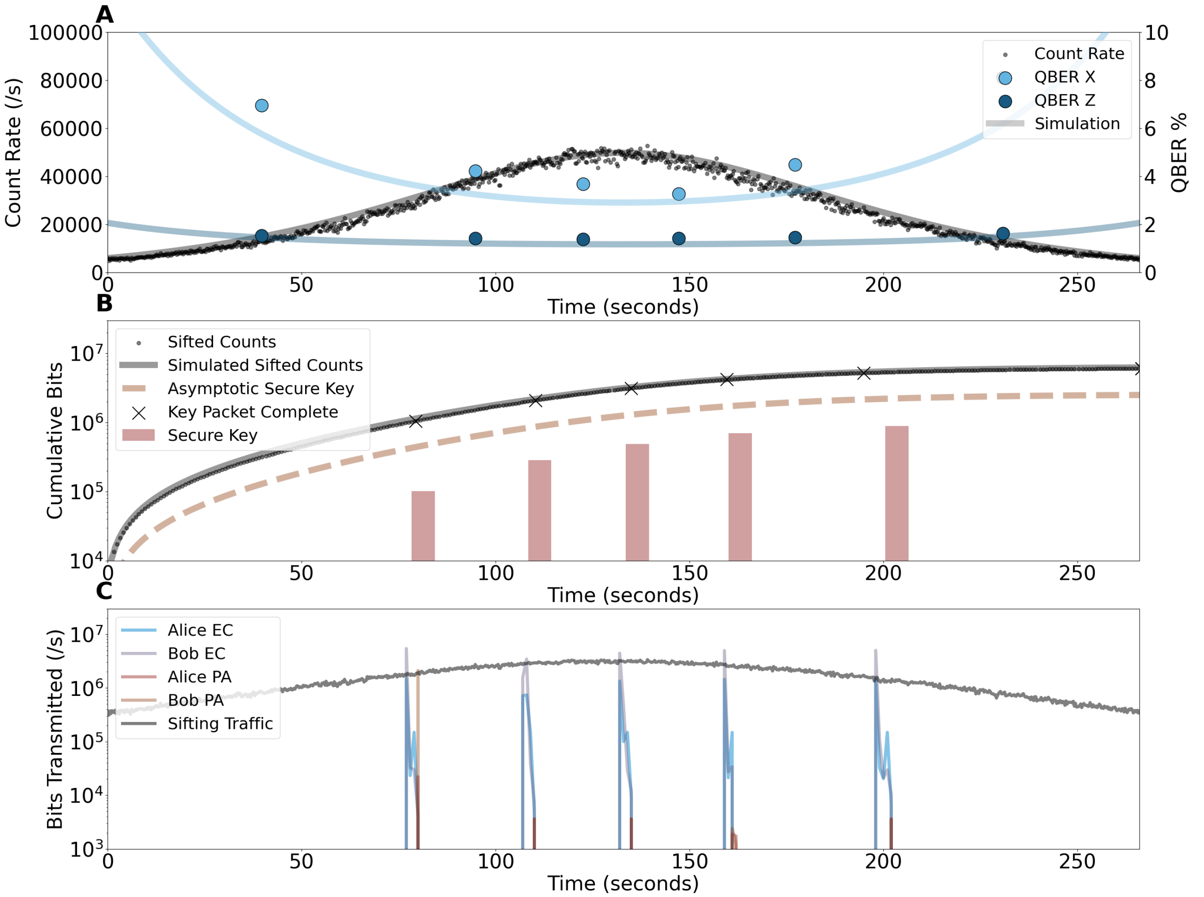

Figure S7: 45° **maximum elevation overpass.** (**A**) Count rate measured at the 4 QRx detectors as a function of the emulated satellite overpass time t_{pass}. The measured data is plot alongside the simulated count rate. The QBER, averaged over a key packet (1 Mbit) is plot (circles) along with the expected QBER from a model which uses the measured system parameters. (**B**) The cumulative measured counts for the emulated overpass are plot. The cumulative asymptotic key can be compared (dashed line) to the cumulative distilled key (bars) when the block size of the PA is set to 1 Mbit= 1 key packet. (**C**) Traffic on the classical communications channel is shown for the sifting process (grey) and the EC & PA processes in each direction.

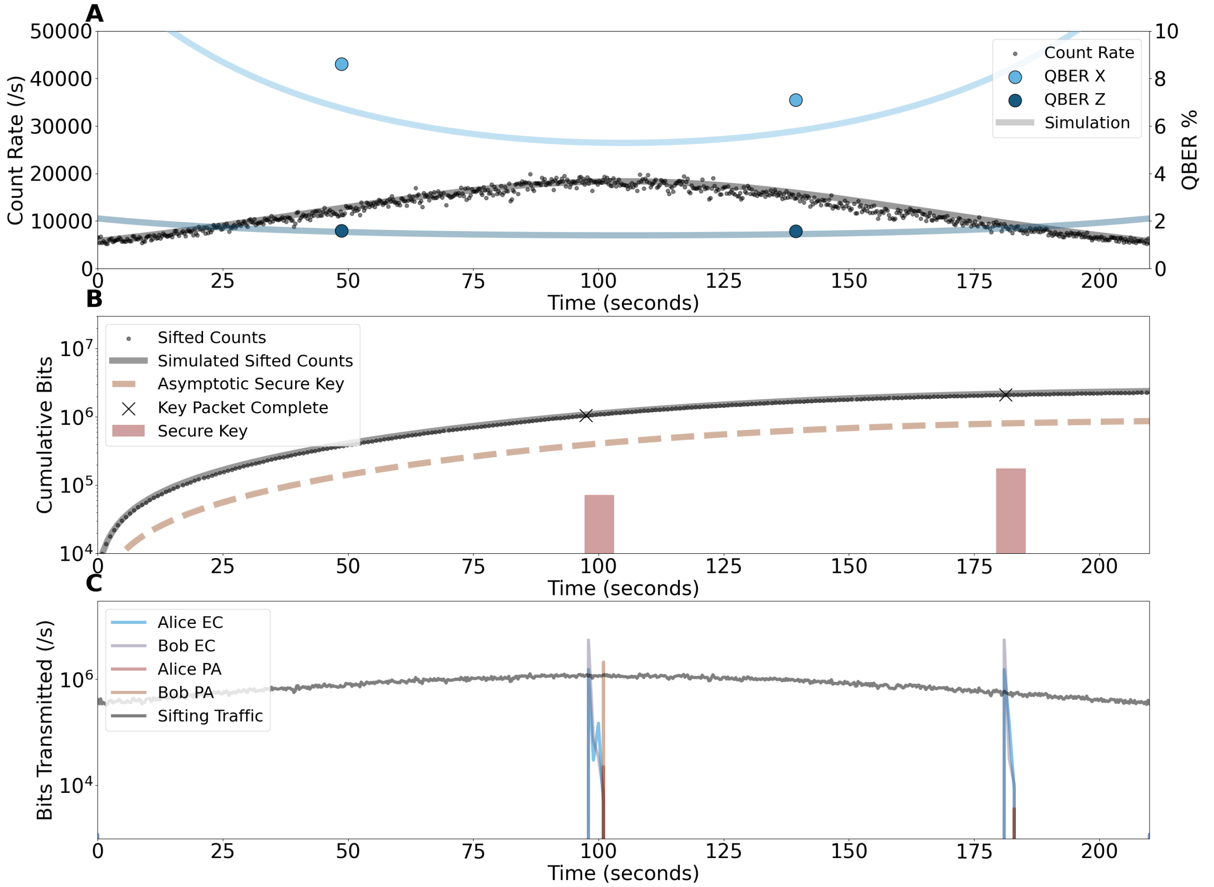

Figure S8: **30° maximum elevation overpass.** (**A**) Count rate measured at the 4 QRx detectors as a function of the emulated satellite overpass time t_{pass}. The measured data is plot alongside the simulated count rate. The QBER, averaged over a key packet (1 Mbit) is plot (circles) along with the expected QBER from a model which uses the measured system parameters. (**B**) The cumulative measured counts for the emulated overpass are plot. The cumulative asymptotic key can be compared (dashed line) to the cumulative distilled key (bars) when the block size of the PA is set to 1 Mbit = 1 key packets. (**C**) Traffic on the classical communications channel is shown for the sifting process (grey) and the EC & PA processes in each direction.

REFERENCES AND NOTES

1. J. F. Dynes, A. Wonfor, W. W. S. Tam, A. W. Sharpe, R. Takahashi, M. Lucamarini, A. Plews, Z. L. Yuan, A. R. Dixon, J. Cho, Y. Tanizawa, J. .P. Elbers, H. Greißer, I. H. White, R. V. Penty, A. J. Shields, *Cambridge quantum network*. *Npj Quantum Inf.* **5**, 101 (2019).
2. M. Peev, C. Pacher, R. Alléaume, C. Barreiro, J. Bouda, W. Boxleitner, T. Debuisschert, E. Diamanti, M. Dianati, J. F. Dynes, S. Fasel, S. Fossier, M. Fürst, J. D. Gautier, O. Gay, N. Gisin, P. Grangier, A. Happe, Y. Hasani, M. Hentschel, H. Hübel, G. Humer, T. Länger, M. Legré, R. Lieger, J. Lodewyck, T. Lorünser, N. Lütkenhaus, A. Marhold, T. Matyus, O. Maurhart, L. Monat, S. Nauerth, J. B. Page, A. Poppe, E. Querasser, G. Ribordy, S. Robyr, L. Salvail, A. W. Sharpe, A. J. Shields, D. Stucki, M. Suda, C. Tamas, T. Themel, R. T. Thew, Y. Thoma, A. Treiber, P. Trinkler, R. Tualle-Brouri, F. Vannel, N. Walenta, H. Weier, H. Weinfurter, I. Wimberger, Z. L. Yuan, H. Zbinden, A. Zeilinger, The SECOQC quantum key distribution network in Vienna. *New J. Phys.* **11**, 075001 (2009).
3. M. Sasaki, M. Fujiwara, H. Ishizuka, W. Klaus, K. Wakui, M. Takeoka, S. Miki, T. Yamashita, Z. Wang, A. Tanaka, K. Yoshino, Y. Nambu, S. Takahashi, A. Tajima, A. Tomita, T. Domeki, T. Hasegawa, Y. Sakai, H. Kobayashi, T. Asai, K. Shimizu, T. Tokura, T. Tsurumaru, M. Matsui, T. Honjo, K. Tamaki, H. Takesue, Y. Tokura, J. F. Dynes, A. R. Dixon, A. W. Sharpe, Z. L. Yuan, A. J. Shields, S. Uchikoga, M. Legré, S. Robyr, P. Trinkler, L. Monat, J.-B. Page, G. Ribordy, A. Poppe, A. Allacher, O. Maurhart, T. Länger, M. Peev, A. Zeilinger, Field test of quantum key distribution in the Tokyo QKD network. *Opt. Express* **19**, 10387–10409 (2011).
4. T. Y. Chen, H. Liang, Y. Liu, W. Q. Cai, L. Ju, W. Y. Liu, J. Wang, H. Yin, K. Chen, Z. B. Chen, C. Z. Peng, J. W. Pan, Field test of a practical secure communication network with decoy-state quantum cryptography. *Opt. Express* **17**, 6540–6549 (2009).
5. S. Pirandola, R. Laurenza, C. Ottaviani, L. Banchi, Fundamental limits of repeaterless quantum communications. *Nat. Commun.* **8**, 15043 (2017).
6. M. Takeoka, S. Guha, M. M. Wilde, Fundamental rate-loss tradeoff for optical quantum key distribution. *Nat. Commun.* **5**, 5235 (2014).
7. R. Bedington, J. M. Arrazola, A. Ling, Progress in satellite quantum key distribution. *Npj Quantum Inf.* **3**, 30 (2017).
8. W. K. Wootters, W. H. Zurek, A single quantum cannot be cloned. *Nature* **299**, 802–803 (1982).
9. H.-J. Briegel, W. Dür, J. I. Cirac, P. Zoller, Quantum repeaters: The role of imperfect local operations in quantum communication. *Phys. Rev. Lett.* **81**, 5932 (1998.

10. L. M. Duan, M. D. Lukin, J. I. Cirac, P. Zoller, Long-distance quantum communication with atomic ensembles and linear optics. *Nature* **414**, 413–418 (2001).
11. N. Sangouard, C. Simon, H. de Riedmatten, N. Gisin, Quantum repeaters based on atomic ensembles and linear optics. *Rev. Mod. Phys.* **83**, 33–80 (2011).
12. S. Guha, H. Krovi, C. A. Fuchs, Z. Dutton, J. A. Slater, C. Simon, W. Tittel, Rate-loss analysis of an efficient quantum repeater architecture. *Phys. Rev. A* **92**, 022357 (2015).
13. M. K. Bhaskar, R. Riedinger, B. Machielse, D. S. Levonian, C. T. Nguyen, E. N. Knall, H. Park, D. Englund, M. Lončar, D. D. Sukachev, M. D. Lukin, Experimental demonstration of memory-enhanced quantum communication. *Nature* **580**, 60–64 (2020).
14. M. Pittaluga, M. Minder, M. Lucamarini, M. Sanzaro, R. I. Woodward, M. J. Li, Z. Yuan, A. J. Shields, 600-km repeater-like quantum communications with dual-band stabilization. *Nat. Photon.* **15**, 530–535 (2021).
15. L. Zhou, J. Lin, Y. Jing, Z. Yuan, Twin-field quantum key distribution without optical frequency dissemination. *Nat. Commun.* **14**, 928 (2023).
16. H. J. Kimble, The quantum internet. *Nature* **453**, 1023–1030 (2008).
17. S. Pirandola, S. L. Braunstein, Physics: Unite to build a quantum internet. *Nature* **532**, 169–171 (2016).
18. S. K. Liao, W. Q. Cai, J. Handsteiner, B. Liu, J. Yin, L. Zhang, D. Rauch, M. Fink, J. G. Ren, W. Y. Liu, Y. Li, Q. Shen, Y. Cao, F. Z. Li, J. F. Wang, Y. M. Huang, L. Deng, T. Xi, L. Ma, T. Hu, L. Li, N. L. Liu, F. Koidl, P. Wang, Y. A. Chen, X. B. Wang, M. Steindorfer, G. Kirchner, C. Y. Lu, R. Shu, R. Ursin, T. Scheidl, C. Z. Peng, J. Y. Wang, A. Zeilinger, J. W. Pan, Satellite-relayed intercontinental quantum network. *Phys. Rev. Lett.* **120**, 030501 (2018).
19. S.-K. Liao, W.-Q. Cai, W.-Y. Liu, L. Zhang, Y. Li, J.-G. Ren, J. Yin, Q. Shen, Y. Cao, Z.-P. Li, F.-Z. Li, X.-W. Chen, L.-H. Sun, J.-J. Jia, J.-C. Wu, X.-J. Jiang, J.-F. Wang, Y.-M. Huang, Q. Wang, Y.-L. Zhou, L. Deng, T. Xi, L. Ma, T. Hu, Q. Zhang, Y.-A. Chen, N.-L. Liu, X.-B. Wang, Z.-C. Zhu, C.-Y. Lu, R. Shu, C.-Z. Peng, J.-Y. Wang, J.-W. Pan, Satellite-to-ground quantum key distribution. *Nature* **549**, 43–47 (2017).
20. J. Yin, Y. H. Li, S. K. Liao, M. Yang, Y. Cao, L. Zhang, J. G. Ren, W. Q. Cai, W. Y. Liu, S. L. Li, R. Shu, Y. M. Huang, L. Deng, L. Li, Q. Zhang, N. L. Liu, Y. A. Chen, C. Y. Lu, X. B. Wang, F. Xu, J. Y. Wang, C. Z. Peng, A. K. Ekert, J. W. Pan, Entanglement-based secure quantum cryptography over 1,120 kilometres. *Nature* **582**, 501–505 (2020).

21. C.-Z. Wang, Y. Li, W. Q. Cai, W. Y. Liu, S. K. Liao, Synchronization using quantum photons for satellite-to-ground quantum key distribution. *Opt. Express* **29**, 29595–29603 (2021).
22. J. S. Sidhu, T. Brougham, D. McArthur, R. G. Pousa, D. K. L. Oi, Finite key effects in satellite quantum key distribution. *Npj Quantum Inf.* **8**, 18 (2022).
23. N. Hosseinidehaj, A. M. Lance, T. Symul, N. Walk, T. C. Ralph, Finite-size effects in continuous-variable quantum key distribution with gaussian postselection. *Phys. Rev. A* **101**, 052335 (2020).
24. M. Lucamarini, K. A. Patel, J. F. Dynes, B. Fröhlich, A. W. Sharpe, A. R. Dixon, Z. L. Yuan, R. V. Penty, A. J. Shields, Efficient decoy-state quantum key distribution with quantified security. *Opt. Express* **21**, 24550–24565 (2013).
25. M. Lucamarini, J. F. Dynes, B. Fröhlich, Z. Yuan, A. J. Shields, Security bounds for efficient decoy-state quantum key distribution. *IEEE J. Sel. Top. Quant. Electron.* **21**, 6601408 (2015).
26. G. T. van Belle, A. B. Meinel, M. P. Meinel, The scaling relationship between telescope cost and aperture size for very large telescopes. arXiv:2107.09605 [quant-ph] (2021).
27. N. Saeed, A. Elzanaty, H. Almorad, H. Dahrouj, T. Y. al-Naffouri, M. S. Alouini, Cubesat communications: Recent advances and future challenges. *IEEE Commun. Surv. Tutor.* **22**, 1839–1862 (2020).
28. A. Carrasco-Casado, Abhijit Biswas, R. Fields, B. Grefenstette, F. Harrison, S. Sburlan, M. Toyoshima, Optical communication on CubeSats—Enabling the next era in space science. arXiv:1811.03413 [quant-ph] (2017).
29. L. Li, X. Zhang, J. Zhang, X. U. Changzhi, J. Yi, Advanced space laser communication technology on cubesats. *ZTE Commun.* **18**, 45–54 (2021).
30. X. Ma, X. Yuan, Z. Cao, B. Qi, Z. Zhang, Quantum random number generation. *Npj Quantum Inf.* **2**, 16021 (2016).
31. T. Roger, T. Paraiso, I. D. Marco, D. G. Marangon, Z. Yuan, A. J. Shields, Real-time interferometric quantum random number generation on chip. *J. Opt. Soc. Am. B* **36**, B137 (2019).
32. C. Abellán, W. Amaya, M. Jofre, M. Curty, A. Acín, J. Capmany, V. Pruneri, M. W. Mitchell, Ultra-fast quantum randomness generation by accelerated phase diffusion in a pulsed laser diode. *Opt. Express* **22**, 1645–1654 (2014).

33. T. K. Paraïso, T. Roger, D. G. Marangon, I. de Marco, M. Sanzaro, R. I. Woodward, J. F. Dynes, Z. Yuan, A. J. Shields, A photonic integrated quantum secure communication system. *Nat. Photon.* **15**, 850–856 (2021).
34. G. Witvoet, S. Kuiper, A. Meskers. Performance validation of a high-bandwidth fine steering mirror for optical communications, in *International Conference on Space Optics Proceedings*—ICSO 2018 (SPIE, 2019).
35. C. M. Schieler, K. M. Riesing, B. C. Bilyeu, B. S. Robinson, J. P. Wang, W. T. Roberts, S. Piazzola, in *2022 IEEE International Conference on Space Optical Systems and Applications* (*ICSOS*) (IEEE, 2022), pp. 181–185; https://doi.org/10.1109/ICSOS53063.2022.9749714.
36. M. T. Gruneisen, M. L. Eickhoff, S. C. Newey, K. E. Stoltenberg, J. F. Morris, M. Bareian, M. A. Harris, D. W. Oesch, M. D. Oliker, M. B. Flanagan, B. T. Kay, J. D. Schiller, R. N. Lanning, Adaptive-optics-enabled quantum communication: A technique for daytime space-to-earth links. *Phys. Rev. Applied* **16**, 014067 (2021).
37. Q. Shen, J. Y. Guan, T. Zeng, Q. M. Lu, L. Huang, Y. Cao, J. P. Chen, T. Q. Tao, J. C. Wu, L. Hou, S. K. Liao, J. G. Ren, J. Yin, J. J. Jia, H. F. Jiang, C. Z. Peng, Q. Zhang, J. W. Pan, Experimental simulation of time and frequency transfer via an optical satellite-ground link at 10-18 instability. *Optica* **8**, 471–476 (2021).
38. H. Dai, Q. Shen, C. Z. Wang, S. L. Li, W. Y. Liu, W. Q. Cai, S. K. Liao, J. G. Ren, J. Yin, Y. A. Chen, Q. Zhang, F. Xu, C. Z. Peng, J. W. Pan, Towards satellite-based quantum-secure time transfer. *Nat. Phys.* **16**, 848–852 (2020).
39. P. Zhang, D. K. L. Oi, D. Lowndes, J. G. Rarity, Timing and synchronisation for high-loss free-space quantum communication with hybrid de bruijn codes. *IET Quant. Comm.* **2**, 80–89 (2021).
40. P. Arteaga-Díaz, D. Cano, V. Fernandez, Practical side-channel attack on free-space QKD systems with misaligned sources and countermeasures *IEEE Access* **4**, 1–11 (2022).
41. J. G. Rarity, P. R. Tapster, P. M. Gorman, P. Knight, Ground to satellite secure key exchange using quantum cryptography. *New J. Phys.* **4**, 82 (2002).
42. D. Dequal, L. Trigo Vidarte, V. Roman Rodriguez, G. Vallone, P. Villoresi, A. Leverrier, E. Diamanti, Feasibility of satellite-to-ground continuous-variable quantum key distribution. *npj Quantum Inf* **7**, 3 (2021).

43. V. M. Acosta, D. Dequal, M. Schiavon, A. Montmerle-Bonnefois, C. B. Lim, J.-M. Conan, E. Diamanti, Analysis of satellite-to-ground quantum key distribution with adaptive. arXiv:2111.06747 [quant-ph] (2021).

44. L. Mazzarella, C. Lowe, D. Lowndes, S. K. Joshi, S. Greenland, D. McNeil, C. Mercury, M. Macdonald, J. Rarity, D. K. L. Oi, Quarc: Quantum research Cubesat—A constellation for quantum communication. *Cryptography* **4**, 7 (2020).

45. D. Vasylyev, W. Vogel, F. Moll, Satellite-mediated quantum atmospheric links. *Phys. Rev. A* **99**, 053830 (2019).

46. I. Ali, N. Al-Dhahir, J. E. Hershey, Predicting the visibility of leo satellites. *IEEE Trans. Aerosp. Electron. Syst.* **35**, 1183–1190 (1999).

47. G. Brassard, L. Salvail, Secret-key reconciliation by public discussion, in EUROCRYPT '93: *Workshop on the Theory and Application of Cryptographic Techniques on Advances in Cryptology* (Springer, Berlin, Heidelberg, 1993), pp. 410–423.

48. J.-P. Bourgoin, E Meyer-Scott, B L Higgins, B. Helou, C. Erven, H. Hübel, B. Kumar, D. Hudson, I D'Souza, R. Girard, R. Laflamme, T. Jennewein, A comprehensive design and performance analysis of low earth orbit satellite quantum communication, *New J. Phys.* **15**, 023006 (2013).

49. C. Bonato, A. Tomaello, V. D. Deppo, G. Naletto, P. Villoresi, Feasibility of satellite quantum key distribution, *New J. Phys.* **11**, 045017 (2009).

50. T. Roger, C. Perumangatt, R. Singh, D. G. Marangon, M. Sanzaro, P. R. Smith, A. J. Shields, in *Proceedings of the SPIE 12446, Quantum Computing, Communication, and Simulation III* (SPIE, 2023), p. 124460K.

51. A. Lee, A. T. Castillo, C. Whitehill, R. Donaldson, Quantum bit error rate timing jitter dependency on multi-mode fibers, *Opt. Express* **31**, 6076–6087 (2023).
